# Quantitative investigation of the direct interaction between Hemagglutinin and fusion proteins of *Peste des petits ruminant* virus using surface Plasmon resonance

**DOI:** 10.1186/s12985-018-0933-7

**Published:** 2018-01-22

**Authors:** Xuelian Meng, Ruixue Deng, Xueliang Zhu, Zhidong Zhang

**Affiliations:** State Key Laboratory of Veterinary Etiological Biology, Lanzhou Veterinary Research Institute, Chinese Academy of Agricultural Sciences, Xujiaping 1, Yanchangpu, Chengguan District, Lanzhou, 730046 China

**Keywords:** Interactions, Hemagglutinin, Fusion protein, Expression, SPR, *Peste des petits ruminanst* virus

## Abstract

**Background:**

The specific and dynamic interaction between the hemagglutinin (H) and fusion (F) proteins of morbilliviruses is a prerequisite for the conformational rearrangements and membrane fusion during infection process. The two heptad repeat regions (HRA and HRB) of F protein are both important for the triggering of F protein.

**Methods:**

In this study, the direct interactions of *Peste des petits ruminants* virus (PPRV) H with F, HRA and HRB were quantitatively evaluated using biosensor surface plasmon resonance (SPR).

**Results:**

The binding affinities of immobilized pCMV-HA-H (HA-H) interacted with proteins pCMV-HA-F (HA-F) and pCMV-HA-HRB (HA-HRB) *(K*_D_ = 1.91 × 10^− 8^ M and 2.60 × 10^− 7^ M, respectively) reacted an order of magnitude more strongly than that of pCMV-HA-HRA (HA-HRA) and pCMV-HA-Tp IGFR-LD (HA) *(K*_D_ = 1.08 × 10^− 4^ M and 1.43 × 10^− 4^ M, respectively).

**Conclusions:**

The differences of the binding affinities suggested that HRB is involved in functionally important intermolecular interaction in the fusion process.

## Background

Entry processes of enveloped viruses are complex and involve a variety of proteins of virus itself and host. In morbilliviruses, two viral glycoproteins plays the key role in the infection process: the hemagglutinin (H) and fusion (F) proteins [1]. The H protein is responsible for binding to the target cell, while the F protein mediates membrane fusion, inducing the virus-cell and cell-cell fusion [2, 3]. To be fusogenically active, F protein must be cleaved from the biologically inactive precursor (F0) to two fusogenically active metastable prefusion fragments: a membrane-anchored F1 and a disulfide-linked F2 subunit [1, 4]. The F1 subunit has several domains: (1) a N-terminus hydrophobic fusion peptide (FP), (2) two heptad repeat regions (HRA and HRB), (3) a transmembrane (TM) domain, and (4) a C-terminal cytoplasmic tail [5, 6]. Although the process of F protein mediated membrane fusion promoted by the H protein is not precisely known, it is appreciated that fusion is induced through a series of conformational changes of F protein that has been triggered by specific interaction with the homologous H protein [1, 7–16], and the HRA, HRB are all important for the triggering of F protein [17–19]. Upon triggering, the heptad repeat regions form a stable six-helix bundle (6-HB), a process intimately linked to membrane fusion [20].

*Peste des petits ruminants* virus (PPRV), a member of the genus Morbillivirus in the Paramyxoviridae family causes an acutecontagious disease. Recently, major disease events herald an epidemic direction, from west to east [21]. *Peste des petits ruminants* (PPR) has recently been targeted as the next candidate for global eradication following Rinderpest by the World Organization for Animal Health (OIE) and the Food and Agriculture Organization (FAO). Thus, quantifying the binding affinity of H and HRA, HRB of F protein interaction is of prime importance to better understand their roles in disease induction as well as in developing the therapeutic drugs and control strategies.

Quantifying the binding affinity of protein-protein interactions in vitro is the basis for studying the biochemical processes. To date, various techniques have been used to assess the interaction of protein pairs. Surface plasmon resonance (SPR) is a widely accepted label free biophysical tool in order to investigate biomolecular interactions in real time [22–32]. SPR can accurately provide data on the affinity, specificity, and kinetic constants (k_a,_ the association rate parameter; k_d,_ the dissociation rate parameter) of biomolecular interactions directly obtained from sensorgrams in few minutes [24, 26, 33, 34]. Therefore, the technology should become more accessible and its applications more diverse [35–37].

In this study, we aimed to quantitatively assess the binding affinity and kinetic characterization between PPRV H and F, HRA and HRB using biosensor surface plasmon resonance. As these proteins mainly expressed in the form of inclusion body in prokaryotic expression system, so the first stage of our study was purifying the proteins expressed in eukaryotic cells. The purification of the recombinant proteins were carried out by co-immunoprecipitation (Co-IP) kit and anti-HA monoclonal antibody. Finally, we examined the binding affinity and kinetics of the interaction of H with F, HRA and HRB.

## Methods

### Plasmids, cell and reagents

The recombinant plasmids pET30a-H (GenBank Accession No. X74443), pCAGGS-F (GenBank Accession No. X74443) and vector pCMV-HA were provided by the Lanzhou Veterinary Research Institute of Chinese Academy of Agricultural Sciences and were used to construct eukaryotic expressing plasmids. CHO-K1 cells were obtained from Shanghai Institutes for Biological Sciences (SIBS). *E. coli* DH5α, T4 DNA ligase and all restriction enzymes were purchased from TaKaRa. The QIAprep® spin miniprep kit was from QIAGENE. F12 K, G418, OPTI-MEM medium and Lipofectamine3000 were products of Invitrogen. FCS was purchased from Gibco BRL Life Tech. Co-IP kit was purchased from Thermo Fisher. Mouse anti-HA monoclonal antibody, isotype control antibody, lexa Fluor 488/HRP-conjugated anti-mouse IgG and 3,30-diaminobenzidine tetrahydrochloride (DAB) were purchased from Sigma. Immobilon-P transfer membranes were purchased from Millipore. CM5 sensor chip, amine coupling kit and all solutions were also purchased from GE Healthcare.

### Construction of eukaryotic expression vectors

The gene encoding H was amplified from pET30a-H and subcloned into pCMV-HA between *Sfi* I and *Kpn* I sites to produce the plasmid pCMV-HA-H (HA-H). The gene encoding F, HRA and HRB were amplified from pCAGGS-F and subcloned into pCMV-HA between *Bgl* II and *Not* I to produce the plasmid pCMV-HA-F (HA-F), pCMV-HA-HRA (HA-HRA) and pCMV-HA-HRB (HA-HRB). The constructs HA-H, HA-F, HA-HRA and HA-HRB contained the upstream sequences for HA tag. The constructs were verified by restriction analysis and DNA sequencing (TaKaRa, Dalian, China). According to previous study, HA tag barely interferes the structure and bioactivities of recombinant protein.

### Cell culture and transfection

The CHO-K1 cells were cultured in a six-well plate at a density of 1 × 10^6^ cells in F12 K supplemented with 5% FCS, 100 U/mL penicillin and 100 U/mL streptomycin. When the cells were 80% confluent, medium was removed and cells were washed with phosphate-buffered saline (PBS). Five microliters of Lipofectamine 3000 (2 mg/mL) and 4 μg DNA were mixed in 250 mL OPTI-MEM medium and incubated for 30 min at room temperature. The mix was then added to the cells and the plate was incubated at 37 °C with 5% CO2 for 4 h. The transfection mix was then removed and 2 mL of complete DMEM/F12 was added and incubated for 48 h.

### Expression and identification of the recombinant proteins

At 48 h post-transfection, cells were washed twice with PBS and divided into two parts. One part was fixed by 4% paraformaldehyde for 30 min at room temperature. After washing three times in PBS, cells were permeabilized by incubation with 0.2% Triton-100 in PBS for 10 min at 4 °C. The cells were blocked with 5% bovine serum albumin in PBS for 1 h at 37 °C after washing three times in PBS. The cells were then incubated with the mouse anti-HA monoclonal antibody (MAb) in PBS for 1 h at 37 °C, respectively. This was followed by washing three times in PBS, and the cells were then incubated with donkey anti-mouse IgG-Alexa Fluor 488 conjugate at 37 °C for 1 h. The cells were washed again and then observed under fluorescence microscope (Olympus).

The cells of the other part were trypsinized and fixed, permeabilized, blocked and processed with anti-HA MAb and anti-mouse IgG-Alexa Fluor 488 conjugate as described above. Cells were washed and resuspended gently in 500 μL PBS and were analyzed by flow cytometry (FACSAria 11, BD, USA). The transfected cells, which were treated with isotype control primary antibody, served as controls. Approximately 1000 cells were used for each analysis. Data was analyzed by Flowjo software.

### Preparation of proteins

CHO-K1 cells were expanded in F12 K culture medium and transfected with recombinant plasmids. At 48 h post-transfection, the cells were harvested and washed two times with ice-cold PBS, and then lysed with lysis buffer 30 minnutes on ice. Cell lysate was collected at 4 °C and centrifuged at 12000 g for 10 min. The recombinant proteins in cell lysate were purified by Co-IP kit and anti-HA monoclonal antibody. The purity was tested with SDS-PAGE electrophoresis and bromophenol blue dyeing.

### SPR study

All experiments were performed at 25 °C using a Biacore™ 3000 instrument and CM5 biosensor chips (Uppsala, Sweden). Kinetic analyses were performed using deafualt settings of Biacore 3000 and recommended SOP walkthrough of CM5 sensorchip. A CM5 sensor chip with carboxymethylated dextran covalently attached on the gold surface was first primed three times with HBS-EP running buffer (10 mM HEPES, pH 7.4, 150 mM NaCl, 3 mM ethylenediaminetetraacetic acid and 0.005% (*v*/v) of P20 surfactant) at a flow rate of 10 mL/min. Flow cell 1 (FC1) was used as the reference flow cell, which was unmodified and lacked the ligand. Flow cell 2 (FC2) was used for immobilization of protein.

Optimal pH value is the decisive factor that determines the immobilization of protein to the CM5 chip surface. Therefore, Immobilization buffer for immobilizing HA-H was first selected separately using the pH scouting procedure, as described in the instrument protocol, using 10 mM sodium acetate buffers pH 4.0, 4.5, 5.0 and 5.5. The protein was solubilized at a final concentration of 20 μg/mL. Each of these solutions (60 μL) was individually injected into the sensor at a flow rate of 10 μL/min. After each sample application was complete, 50 mM NaOH was used to clean the sample loop in accordance with the manufacturer’s instructions. To control the nonspecific interactions, we performed the same experiment with pCMV-HA-Tp IGFR-LD (HA): the extracellular domain of Taenia pisiformis insulin-like growth factor receptor, at the flow cell 2 of the sensor chip. Each step of the immunoassay was injected at a flow rate of 10 μL/min.

The protein HA-H diluted in 10 mM sodium acetate at the optimal pH was covalently coupled to a CM5 chip with a standard amine-immobilization kit. The carboxyl acid functional groups on the sensor chip surface was activated by 1-ethyl-3-(3-dimethylaminopropyl)carbodiimide hydrochloride (EDC, 0.4 M) and N-hydroxysuccinimide (NHS, 0.1 M) (1:1, *v*/v) and 20 μg/mL HA-H was immobilized on the sensor surface. The remaining NHS ester groups were blocked by 1 M ethanolamine for 10 min.

To investigate the interaction of HA-H with HA-F, HA-HRA and HA-HRB, the experiment was repeated with different concentrations of analytes (12.5, 25, 50,100 and 200 nM). At the end of the dissociation period of each experiment corresponding to one specific concentration of analytes, the sensor chip was regenerated to remove any remaining bound material with a 30 s pluse of 10 mM glycine–HCl (pH 2.5) at 20 μL/min for subsequent usages. The sensorgrams and measurements for interactions of protein-protein were recorded in real time. Responses were measured in RUs as the difference between active and reference channel. For BIAcore instruments, 1 RU corresponds to 1 pg/mm^2^.

### Data analyses

Association and dissociation rate constants (k_a_ and k_d,_ respectively) and the equilibrium dissociation constant (K_D_, k_d_/k_a_) were obtained by fitting of both the association and dissociation phases for HA-F, HA-HRA and HA-HRB to a single-site binding model (1:1 L binding) with mass transfer limitations for determination of the binding kinetics. Data were analyzed with the BIA evaluation software 4.1 (GE Healthcare, Inc., Piscataway, NJ).

## Results

### Expression and identification of the recombinant proteins

**Fig. 1:**
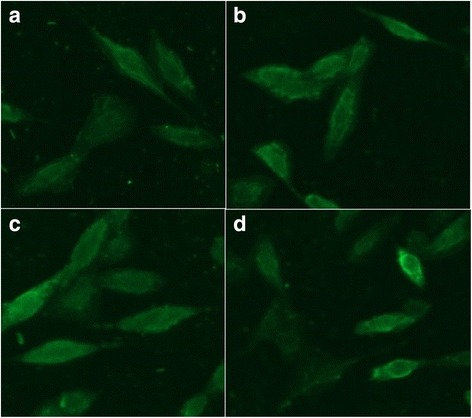
Indirect immunofluorescent assay of expression of recombinant plasmids. **a** HA-H transfected cells. **b** HA-F transfected cells. **c** HA-HRA transfected cells. **d** HA-HRB transfected cells

**Fig. 2:**
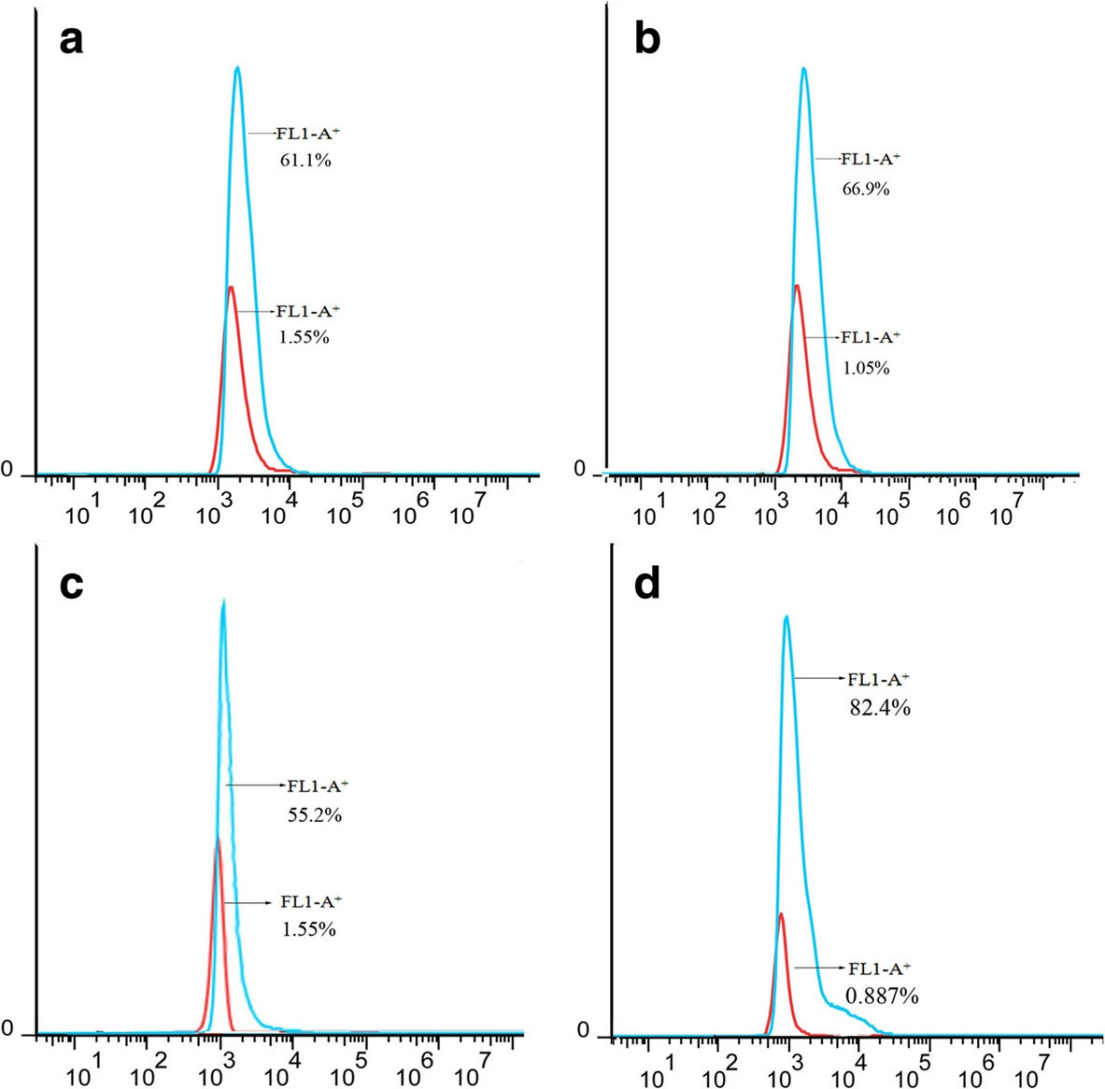
Flow cytometric analyses of expression of recombinant plasmids. **a** HA-H transfected cells. **b** HA-F transfected cells. **c** HA-HRA transfected cells. **d** HA-HRB transfected cells

**Fig. 3:**
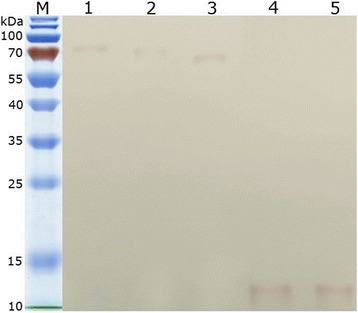
Western blot analysis of the recombinant proteins which were expressed in cells. Lane M: the 10 kDa protein ladder; Lane 1: HA-H protein; Lane 2: HA-F protein; Lane 3: HA protein; Lane 4: HA-HRA protein; Lane 5: HA-HRB protein

The amplified gene encoding PPRV H, F, HRA and HRB were digested by restriction enzymes, and directional cloned into pCMV-HA plasmid to generate successfully the HA fusion expression vectors HA-H, HA-F, HA-HRA and HA-HRB, respectively. To determine whether recombinant plasmids could express the recombinant proteins with HA tag, an immunofluorescence assay, flow cytometric analysis and western blot analysis were performed. As shown in Fig. 1, fluorescence signal was observed in the plasmids-transfected cells. In contrast, no fluorescence signal was observed in the non-transfected cells. The positive proportions and median of fluorescence intensity of plasmids-transfected CHO cells treated with anti-HA MAb were far higher than that of cells treated with isotype control antibody (Fig. 2). All recombinant proteins with expected molecular weights were expressed in the transformed cells (Fig. 3). The above results confirmed that the recombinant proteins were successfully expressed in cells.

### Preparation of the recombinant proteins

**Fig. 4:**
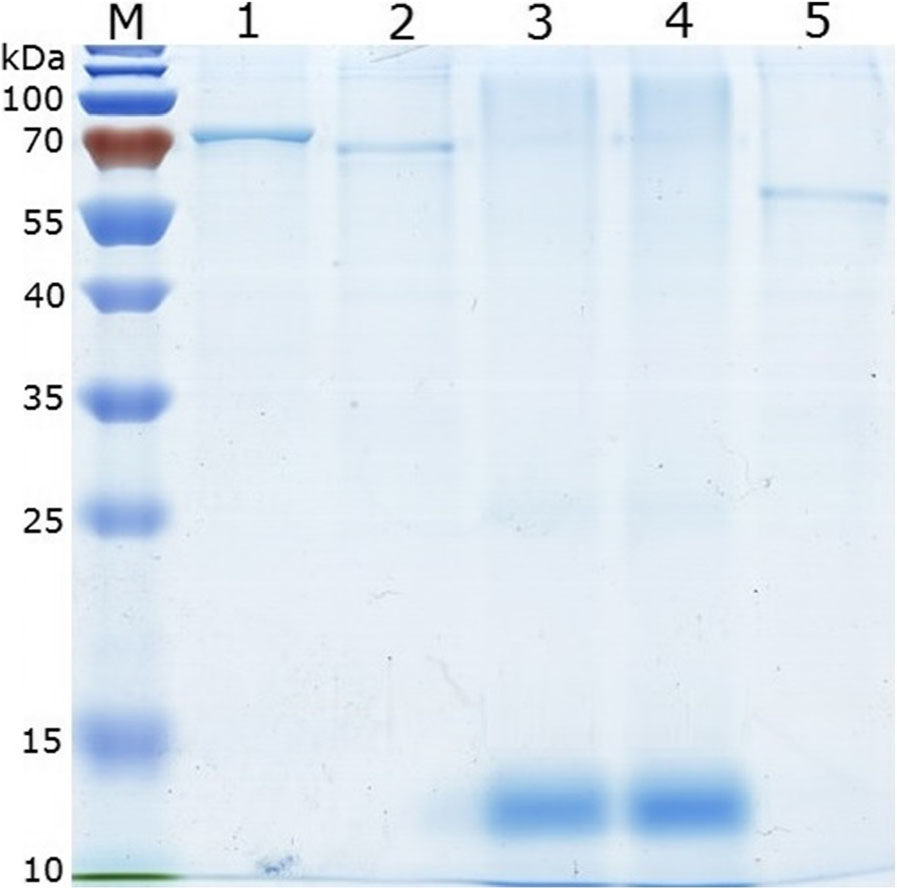
SDS-PAGE analysis of the recombinant proteins which were purified. Lane M: the 10 kDa protein ladder; Lane 1: HA-H protein; Lane 2: HA-F protein; Lane 3: HA-HRA protein; Lane 4: HA-HRB protein; Lane 5: HA protein

CHO-K1 cells were expanded and transfected with recombinant plasmids HA-H, HA-F, HA-HRA and HA-HRB, respectively. At 48 h post-transfection, the cells were harvested, washed and lysed. The recombinant proteins were purified by Co-IP kit and anti-HA monoclonal antibody. The concentrations of the recombinant proteins HA-H, HA-F, HA-HRA, HA-HRB and HA were 233, 331, 243, 315 and 268 μg/mL determined by NanoDrop ND-2000 (Thermo, USA), respectively. The purity of the recombinant proteins were 92.4%, 91.7%, 93.4%, 92.9% and 92.1%, respectively of HA-H, HA-F, HA-HRA, HA-HRB and HA (Fig. 4).

### Preparation of sensor surfaces

**Fig. 5:**
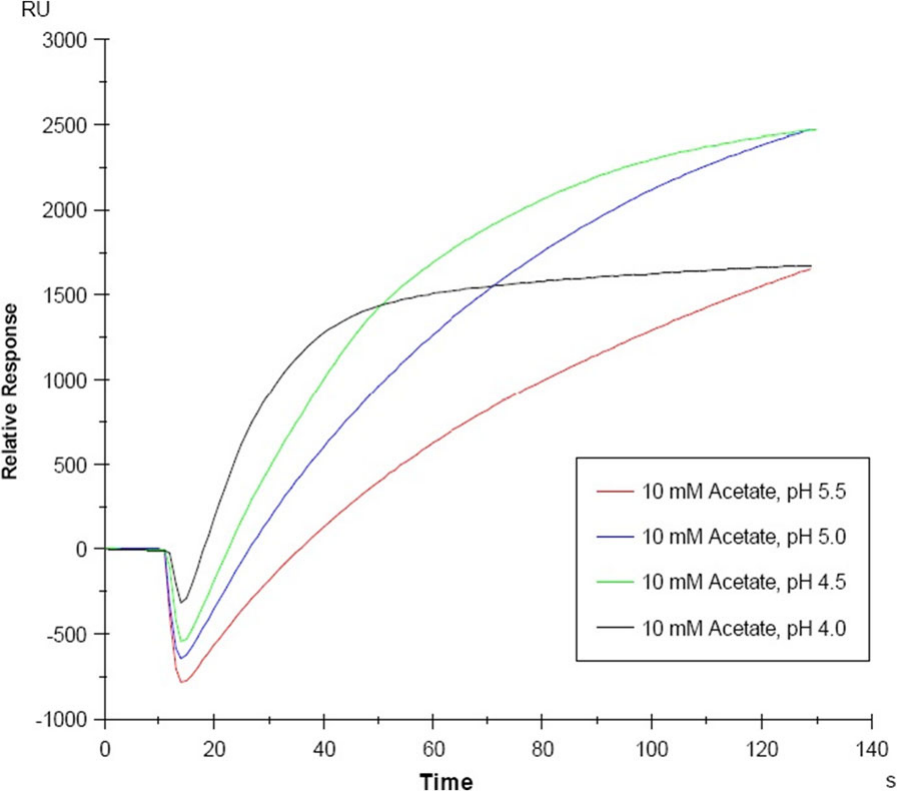
pH optimization for HA-H immobilization on a CM5 sensor chip. 10 mM sodium acetate solutions of pH 4.0, 4.5, 5.0, and 5.5 were used, buffer at pH 4.5 was optimal solution for immobilization

To scout the optimal pH for immobilization of to the CM5 sensor chip, we tested 10 mM sodium acetate solutions of pH 4.0, 4.5, 5.0 and 5.5. Of these, the immobilization buffer selected was 10 mM sodium acetate pH 4.5 provided the best electrostatic interaction of HA-H with the chip surface (Fig. 5). We then used EDC and NHS to activate a CM5 chip surface to initiate covalent coupling of HA-H diluted in 10 mM sodium acetate (pH 4.5) to the active surface. Primary amines on the HA-H surface were then cross-linked to esters on the chip surface to form stable amide bonds. Unbound surface-activated groups were inactivated by 1 M ethanolamine. The expected amount of 6400 RU was detected by Biacore 3000 software.

### Capture assay and binding kinetics

**Fig. 6:**
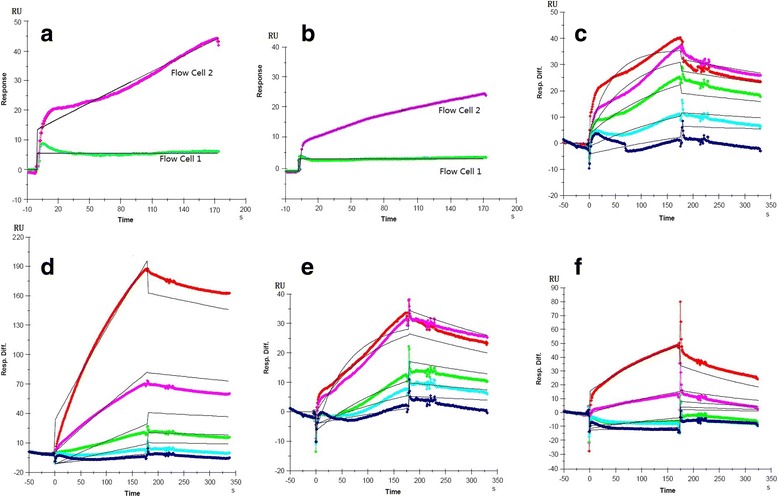
The chip fidelity and sensorgrams of interaction between the immobilized HA-H and purified analytes at different concentrations (12.5, 25, 50, 100 and 200 nM). **a** the sensorgram of HA-F (200 nM) injected into Flow cell 2 and blank runs at Flow cell 1. **b** the sensorgram of HA (200 nM) injected into Flow cell 2 and blank runs at Flow cell 1. **c** HA-F. **d** HA-HRA. **e** HA-HRB. **f** HA

The kinetic study was performed testing different concentrations of purified recombinant proteins against immobilized HA-H. The recombinant proteins were two-fold serially diluted (12.5, 25, 50,100 and 200 nM) with running buffer and then used, involving HA-H immobilized in flow cell 2. Binding results were elaborated independently for each sample. After regeneration, the chip fidelity was sure (Fig. 6a & 6b). The sensorgrams indicated that HA-H interacted strongly with purified recombinant proteins HA-F and HA-HRB, and that the SPR signal increased with increased concentrations of the recombinant proteins (Fig. 6c–f). Little binding was observed HA-H interacted with HA-HRA and HA.

The kinetic parameters (k_a_ and k_d_) and equilibrium dissociation constant (K_D_) were calculated describing the interactions between each purified protein and the immobilized H using the 1:1 L binding model (Table 1). The immobilized H interacted with proteins HA-F, HA-HRA and HA-HRB with k_a_ values of 4.91 × 10 ^4^ M^− 1^·s^− 1^ > k_a_ > 3.82 × 10 M^− 1^·s^− 1^, k_d_ values of 2.99 × 10^− 3^ s^− 1^ > k_d_ > 9.37 × 10^− 4^ s^− 1^. The k_a_ value of H interacted with protein NC was 2.96 × 10 M^− 1^·s^− 1^, k_d_ value of 4.24 × 10^− 3^ s^− 1^.These values indicated a relatively stable complex formed by immobilized HA-H and F, HRB. The K_D_ values for binding of HA-F, HA-HRA and HA-HRB to HA-H were 1.91 × 10^− 8^ M, 1.08 × 10^− 4^ M and 2.6 × 10^− 7^ M, respectively. The K_D_ value for binding of HA was 1.43 × 10^− 4^ M.

**Table 1:** The interaction of immobilized HA-H and purified recombinant proteins

Sample	k_a_ (1/Ms)	k_d_ (1/s)	KD (k_d/_k_a,_ M)
HA-F	4.91 × 10^4^	9.37 × 10^−4^	1.91 × 10^−8^
HA-HRA	3.82 × 10	4.14 × 10^−3^	1.08 × 10^−4^
HA-HRB	1.15 × 10^4^	2.99 × 10^−3^	2.60 × 10^−7^
HA	2.96 × 10	4.24 × 10^−3^	1.43 × 10^−4^

*Note*: kinetic and affinity data were determined by local fitting of the binding step of the individual sensorgrams to a 1:1 L binding model. k_a_: association rate constant; k_d_: dissociation rate constant; K_D_: the equilibrium dissociation constant (k_d_/k_a_)

## Discussion

Decisive interactions for viral tropism occur at the viral entry process. H/ Hemagglutinin-neuraminidase (HN) and F proteins of paramyxovirus are involved in the process. In the past decade, structural biology and biochemistry of H/HN and F proteins of paramyxoviruses have brought new knowledge towards understanding the mechanism of viral membrane fusion. The fusion is induced through a series of conformational changes of F protein triggered by specific interaction with the homologous H/HN protein [1, 9, 38]. In particular, recent studies showed that H/HN-head, −stalk domains and multiple regions of F protein, including HRA and HRB, are critical for the interaction of H/HN protein with the homologous F protein [17–19, 39–48].

Although the interaction between H/HN and F proteins of paramyxovirus had been investigated by Co-IP, IFA and pull-down in previous studies [11, 12, 14, 16, 49–51], the differences of the interaction force between H/HN and F proteins, especially between H/HN and HRA, H/HN and HRB, are still rarely investigated. In order to detect the interaction between proteins by biochemical and biophysical method, it is necessary to prepare soluble proteins. Small affinity tags offer advantages for expression and purification of the recombinant soluble protein. For the study reported herein, we subcloned the genes encoding PPRV H, F, HRA and HRB into pCMV-HA vectors and obtained successfully high purity of soluble HA-H, HA-F, HA-HRA and HA-HRB expressed in the CHO-K1 cells by anti-HA tag antibody and Co-IP kit.

We quantitatively evaluated the interactions of HA-H with HA-F, HA-HRA and HA-HRB using SPR. As a surface-sensitive technique, SPR is ideal for studying interactions between immobilized ligands and analytes because it directly generates reliable kinetic constants (k_a_ and k_d_) from sensorgrams, and produces the reaction within a few minutes, and can analyze both the association and dissociation phases of an interaction, allowing for the detection of weak binding events that would otherwise be difficult to characterize [22, 52]. The RU on the surface is directly indicating the amount of analyte bound. A 1:1 “Langmuir binding” model taken into account the limitations of mass transfer was used to fit the data to determine the binding kinetics. The equilibrium dissociation constant K_D_ is calculated by describing the interactions between the immobilized ligands and analytes. Our SPR data demonstrates direct binding of HA-F, HA-HRA and HA-HRB to HA-H. The HA-F and HA-HRB interacted with the immobilized HA-H at an apparent affinity of 2.60 × 10^− 7^ M > K_D_ > 1.91 × 10^− 8^ M, and that of HA-HRB, as well as HA-F protein, was obviously stronger than that of HA-HRA. The data suggested that PPRV HRB plays more important role than HRA in the viral fusion process. The results were consistent with the previous reports using independent means [5, 41]. Unfortunately, due to the non-open source of Biacore3000 and CM5 chip, this restricts the further investigation of the experimental conditions.

## Conclusions

We constructed an efficient system for the purification of HA-H, HA-F, HA-HRA and HA-HRB expressed in CHO-K1. The real-time SPR characterization of the interactions between HA-H and HA-F, HA-HRA, HA-HRB was determined for the first time. The data of this study clearly demonstrated the high affinity and specific interactions between the immobilized HA-H and HA-F, HA-HRB by reacted an order of magnitude more strongly than that of HA-HRA and HA. This suggests that HRB is most likely involved in functionally important intermolecular interaction in the fusion process.

## Abbreviations

Co-IP: Co-immunoprecipitation
DAB: 3: 30-diaminobenzidine tetrahydrochloride
F: Fusion protein
H: Hemagglutinin
HA: pCMV-HA
HA-F: pCMV-HA-F
HA-H: pCMV-HA-H
HA-HRA: pCMV-HA-HRA
HA-HRB: pCMV-HA-HRB
HN: Hemagglutinin-neuraminidase
ka: The association rate parameter
KD (kd/ka): Equilibrium dissociation constant
kd: The dissociation rate parameter
PPR: *Peste des petits ruminants*
PPRV: *Peste des petits ruminants* virus
SPR: Surface plasmon resonance
